# Supplementary material for: Proteomic analysis of the pyrenoid‐traversing membranes of Chlamydomonas reinhardtii reveals novel components
Source: New Phytol. 2025 Oct 21;249(1):359–72. doi: 10.1111/nph.70669 (PMC12676092; doi:10.1111/nph.70669)
Supplement: Supplementary file 8 — Fig. S1 RBMP1 and RBMP2 localize to different regions of the pyrenoid tubules in Chlamydomonas reinhardtii. Fig. S2 Comparison of LCI16's and PME1's expression profiles in diurnally grown Chlamydomonas reinhardtii cells to the expression profiles of known tubule proteins and Early‐Light‐Induced Proteins (ELIPs). Fig. S3 α‐LCI16 antibody staining in Chlamydomonas reinhardtii is non‐specific but consistent with LCI16 tubule localization. Fig. S4 PCR verification of the mapped insertions in the Chlamydomonas reinhardtii lci16 and pme1 mutants. Fig. S5 Additional TEM images of Chlamydomonas reinhardtii pyrenoids confirm that lci16 and pme1 insertional mutants have normal tubule morphology. Fig. S6 AlphaFold 3 predicts an amphipathic helix and beta sheet in the Chlamydomonas reinhardtii protein PME1. Table S1 Primers used for PCR verification of the Chlamydomonas reinhardtii lci16 and pme1 insertional mutant alleles. Please note: Wiley is not responsible for the content or functionality of any Supporting Information supplied by the authors. Any queries (other than missing material) should be directed to the New Phytologist Central Office. [file NPH-249-359-s007.pdf]

New Phytologist Supporting Information

Article title: **Proteomic analysis of the pyrenoid-traversing membranes of *Chlamydomonas reinhardtii* reveals novel components**

Authors: Eric Franklin, Lianyong Wang, Edward Renne Cruz, Keenan Duggal, Sabrina L. Ergun, Aastha Garde, Alice Lunardon, Weronika Patena, Cole Pacini, Martin C. Jonikas

Article acceptance date: 22 September 2025

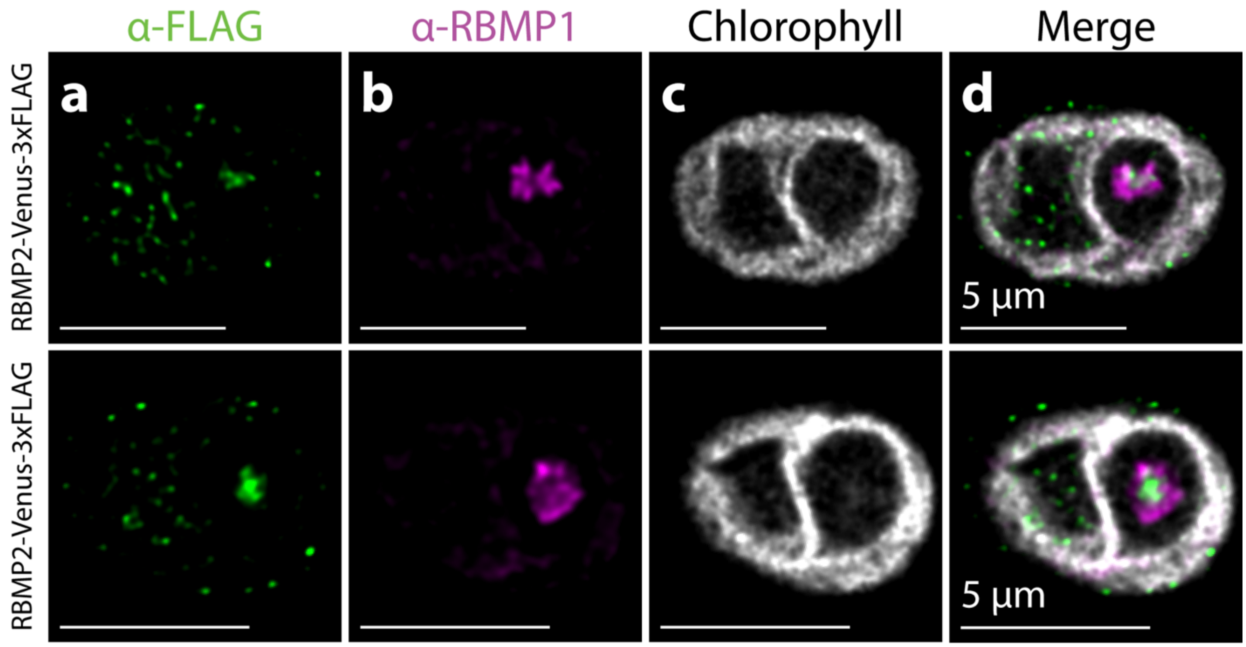

Supporting Information Figure S1

RBMP1 and RBMP2 localize to different regions of the pyrenoid tubules in *Chlamydomonas reinhardtii*. Wild-type cells expressing RBMP2-Venus-3×FLAG were immunostained using α-FLAG **(a)** and α-RBMP1 **(b)** antibodies. Each row represents an independent cell from the same RBMP2-Venus-3×FLAG strain. A gap in the chlorophyll signal **(c)** in the base of each cell (on the right side of each image) represents the location of the pyrenoid. Merging the channels **(d)** shows the distinct localization of RBMP1 and RBMP2.

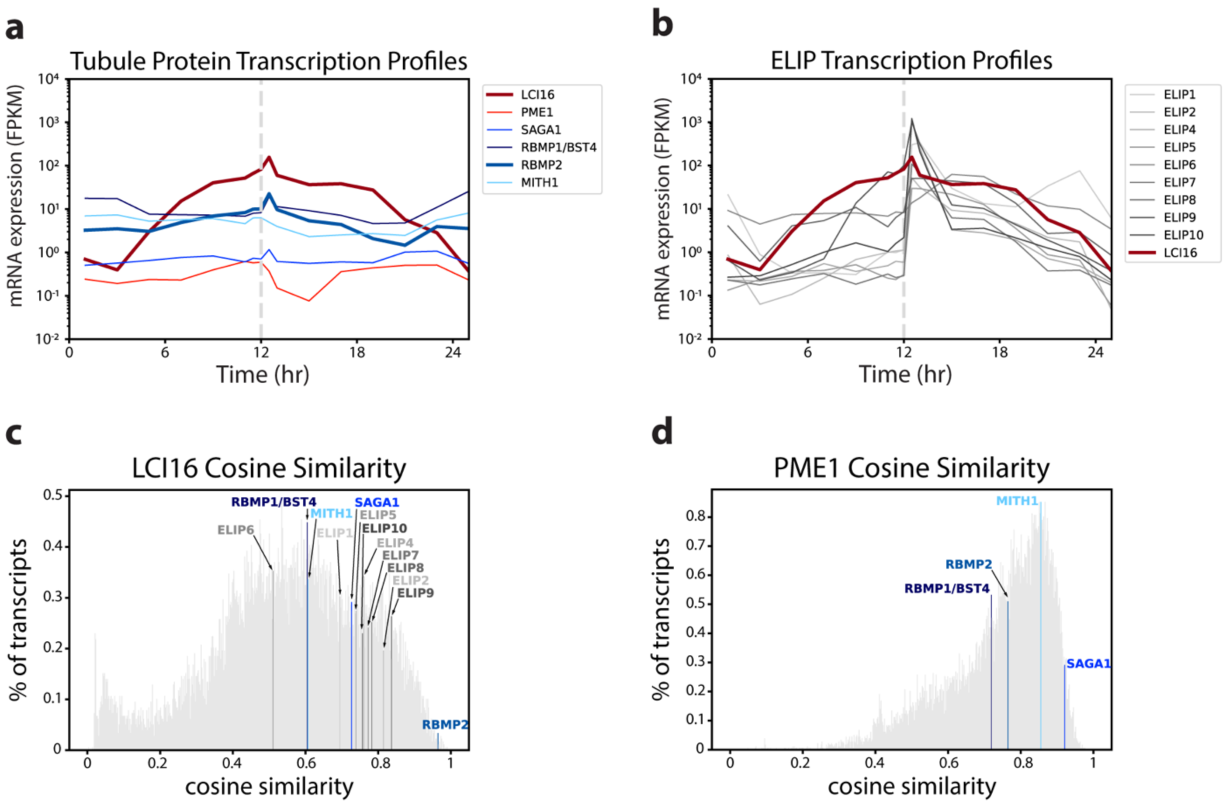

Supporting Information Figure S2

Comparison of LCI16's and PME1's expression profiles in diurnally-grown *Chlamydomonas reinhardtii* cells to the expression profiles of known tubule proteins and ELIP proteins. **a-b.** Plots showing the mRNA abundance, measured in fragments per kilobase million (FPKM), of known pyrenoid tubule proteins (**a**, blue lines) and the Early Light-Induced Proteins (**b**, grey lines) in diurnally-grown *C. reinhardtii* cells (Strenkert *et al.*, 2019). The transcription profile of LCI16 is shown in dark red in both panels. Hours 0 and 24 correspond to the onset of dark and hour 12 corresponds to the onset of light. **c-d.** Histograms showing the cosine similarity of the Strenkert transcriptome profiles to the LCI16 **(c)** and PME1 **(d)** transcriptome profiles. The cosine similarity calculation considers each gene's transcription profile as a 16-dimensional vector (each dimension corresponding to one of the 16 timepoints) and measures the pairwise similarity between transcription profiles by calculating the cosine of the angle between the profiles' 16-D vectors. A cosine similarity of 1 corresponds to vectors that are parallel, i.e. identical transcriptome profiles. Note that because the angle between vectors is dependent on their direction, rather than their magnitude, this metric measures the similarity of the shape of the transcription profiles (i.e., which timepoints are up- and down-regulated), rather than the absolute transcript abundance at each timepoint. Bins containing known tubule proteins or ELIPs are colored as in panels **a-b**.

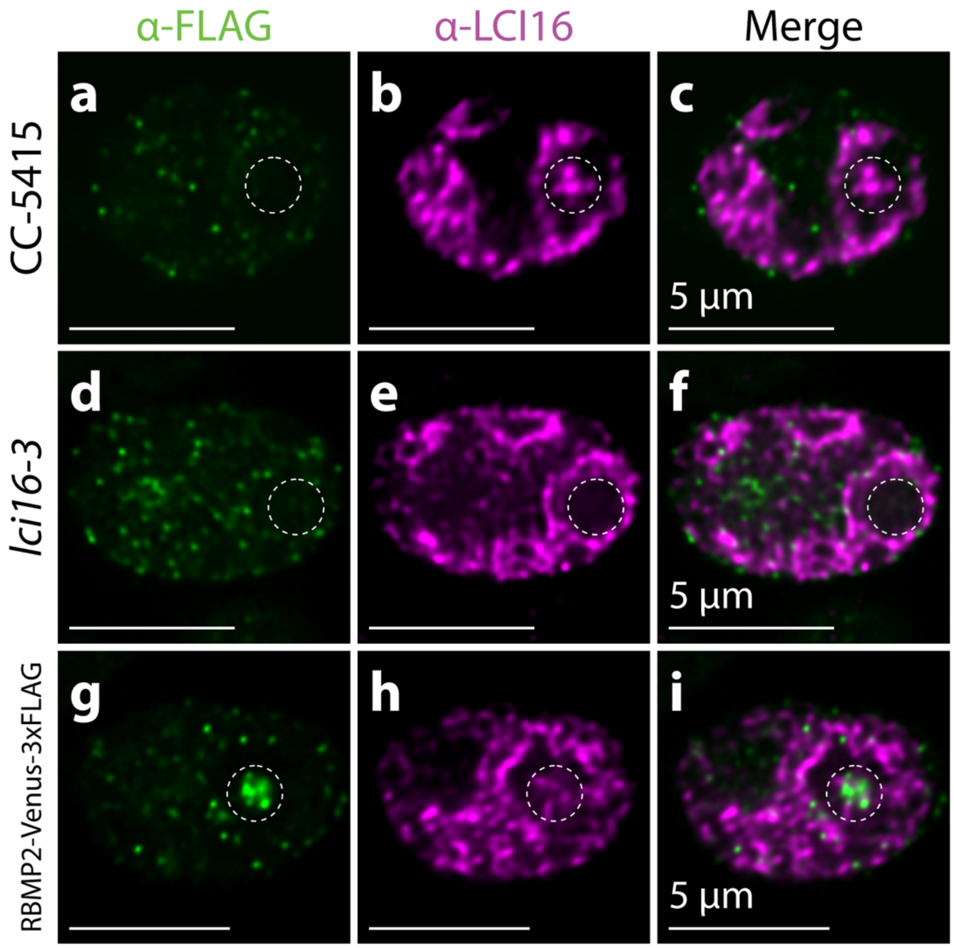

Supporting Information Figure S3

LCI16 antibody staining in *Chlamydomonas reinhardtii* is non-specific but consistent with LCI16 tubule localization. α-FLAG and α-LCI16 immunostaining in wild-type **(a-c)**, *lci16-3* **(d-f)**, and RBMP2-Venus-3×FLAG **(g-i)** cells. Pyrenoids are denoted with dashed circles. The α-LCI16 antibody stains the entire chloroplast, including the pyrenoid, in wild-type **(b)** and RBMP2-Venus-3×FLAG cells **(h)**. However, in the *lci16-3* mutant, the α-LCI16 signal is notably absent from the pyrenoid **(e)**.

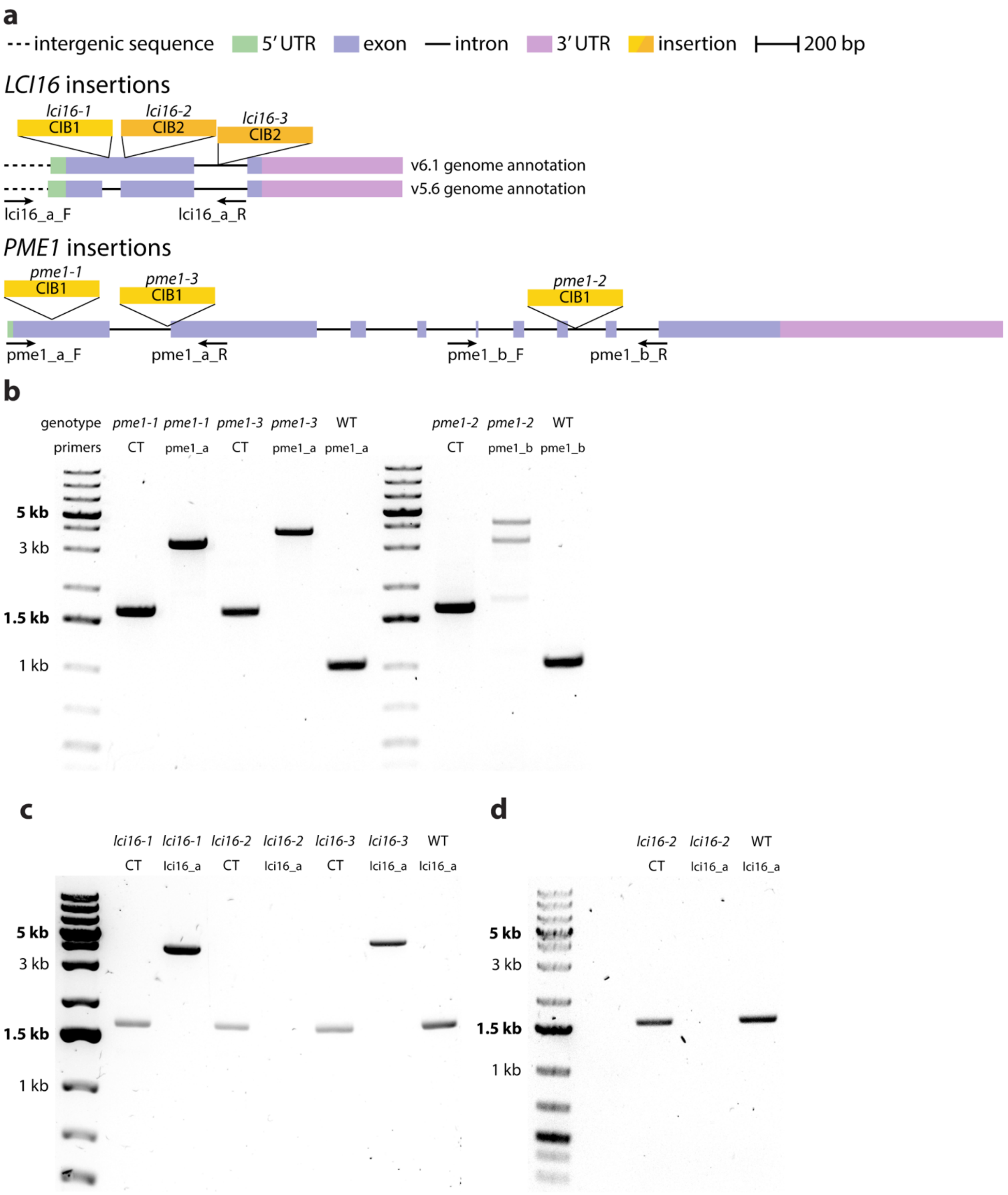

Supporting Information Figure S4

PCR verification of the mapped insertions in the *Chlamydomonas reinhardtii lci16* and *pme1* mutants. **a.** Maps of the LCI16 and PME1 genomic loci showing the locations of the CIB1 and CIB2 insertion cassettes and the primers used for PCR verification. The *lci16-1* insertion falls in

a region that is annotated as an intron in the v5.6 *C. reinhardtii* genome assembly and an exon in the v6.1 assembly—both gene models are shown for clarity. Cassettes and primers are not drawn to scale, but distances between them are to scale. Scale bar 200 bp. **b-d.** Agarose gels showing amplification across the insertion junction in genomic DNA purified from the *pme1* **(b)** and *lci16* **(c-d)** mutants. Expected wild-type band lengths for each primer set can be found in Supporting Information Table **S1**. For all strains except *lci16-2*, band lengths in excess of the expected wild-type amplicon lengths were observed **(b-c)**, indicating the presence of the insertional cassette. The *lci16-2* gDNA failed to amplify using the insertion-spanning primers in panel **(c)**, though the successful amplification using the control (CT) primers confirmed the presence of template gDNA. Panel **(d)** shows that the failure of the lci16_a primer pair to amplify *lci16-2* gDNA in independent PCR reactions is reproducible. Such a reproducible failure to amplify despite a working control is consistent with a very large insertion, and was taken as an indication of a correct insertion, as previously done (Kafri *et al.*, 2023). kb = kilobases.

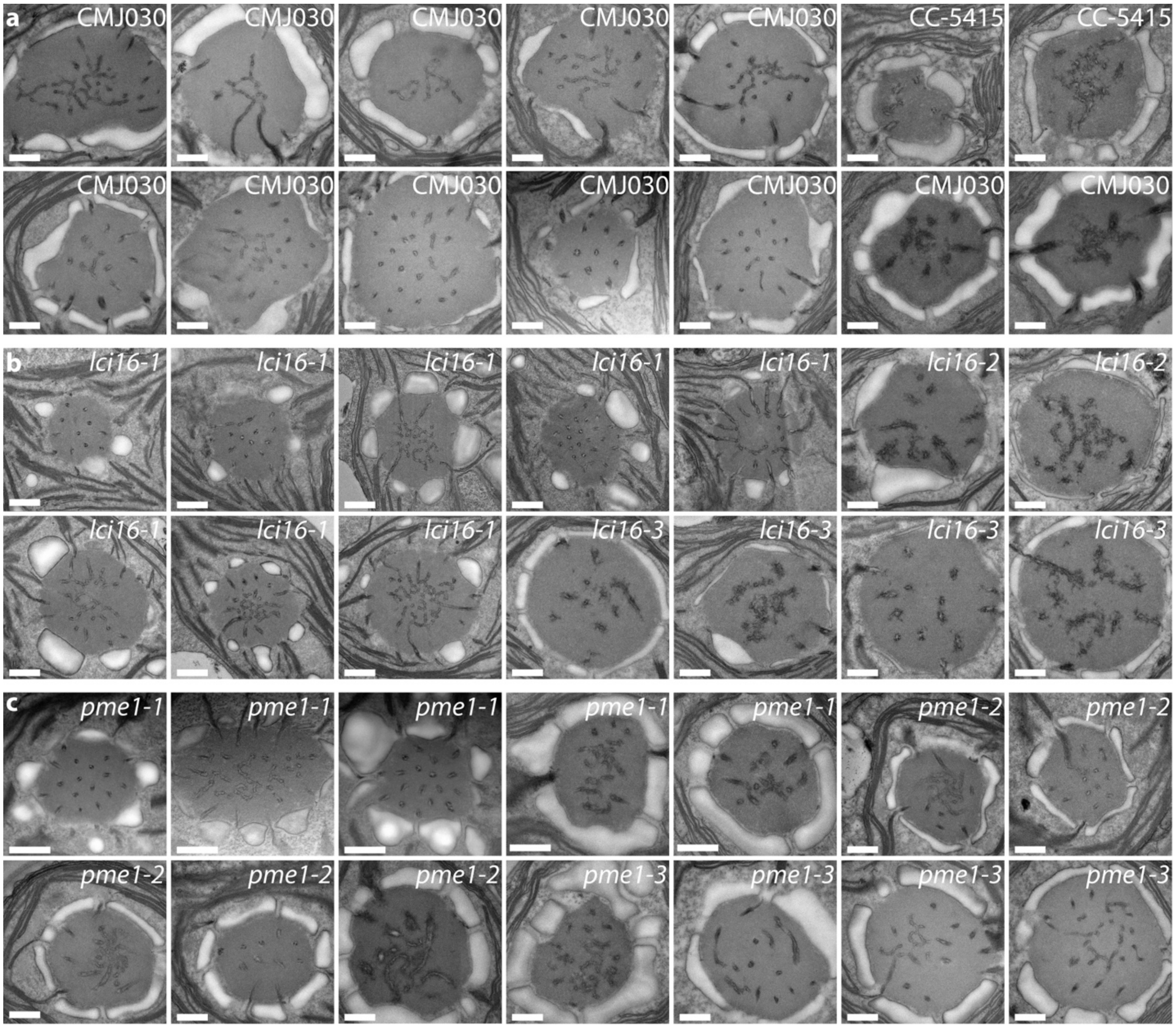

Supporting Information Figure S5

Additional TEM images of *Chlamydomonas reinhardtii* pyrenoids confirm that *lci16* and *pme1* insertional mutants have normal tubule morphology. Fourteen additional micrographs each of wild-type **(a)**, *lci16* **(b)**, and *pme1* **(c)** pyrenoids, representing all mutant alleles and showing that *lci16* and *pme1* mutant pyrenoids contain central reticulated regions and minitubule-containing peripheral tubules. CMJ030 is the parent strain of *lci16-1* and all *pme1* strains, while CC-5415 is the parent strain of *lci16-2* and *lci16-3*. Scale bars 500 nanometers.

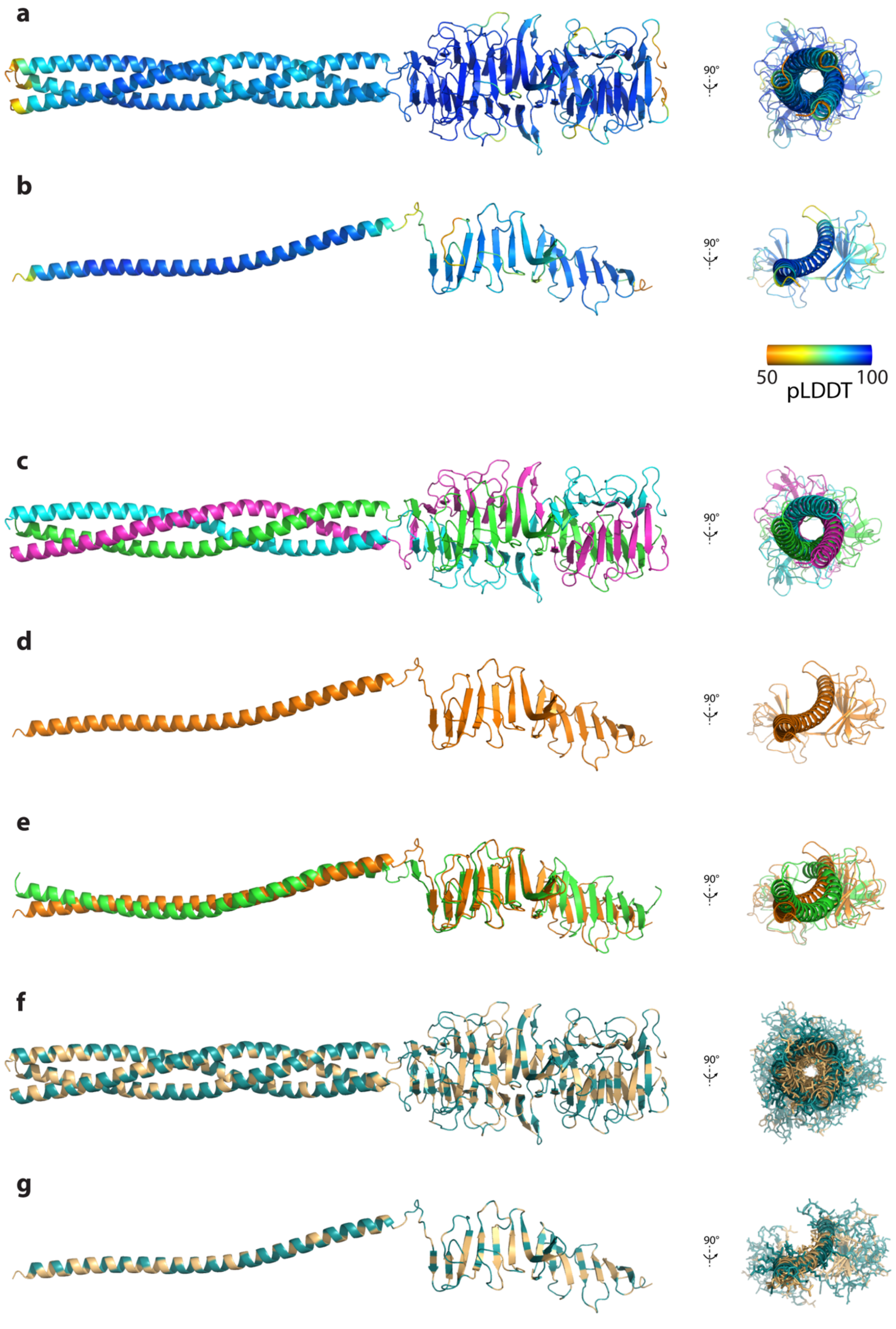
a
90°
b
90°
50
pLDDT
100
c
90°
d
90°
e
90°
f
90°
g
90°

Supporting Information Figure S6

AlphaFold 3 predicts an amphipathic helix and beta sheet in the *Chlamydomonas reinhardtii* protein PME1. Modeled here are a predicted trimer **(a,c,f)** and monomer **(b,d,g)** of PME1 residues 380-639, the 260 residues at its C-terminus. **a-b.** The trimer **(a)** and monomer **(b)** colored according to the per-atom confidence metric pLDDT (predicted local distance difference test). The (interface) predicted template modelling scores for the entire structure are higher for the trimer (pTM = 0.83, iPTM = 0.82) than the monomer (pTM = 0.49). **c-e.** Cartoon representation of the extended helix and beta sheet of the predicted trimer **(c)**, in which the helices form a coiled coil and the beta sheets form a beta-barrel-like structure, and the monomer **(d)**, which takes on a similar conformation even in the absence of oligomerization, as shown by an overlay of the monomer with one subunit of the trimer **(e)**. **f-g.** The predicted trimer **(f)** and monomer **(g)** colored by Kyte-Doolittle hydrophobicity (Kyte & Doolittle, 1982), with hydrophilic residues colored deep teal and hydrophobic residues colored beige. In the 90-degree rotations showing the structure along the axis of the helix, side chain visualization has been added to show that the interior of the coiled coil contains almost exclusively hydrophobic residues, while the exterior is almost entirely hydrophilic.

Primer name	Sequence	Strains tested	Expected amplicon length (bp)
CT_F	CGGTGATACTTACACGCCC	All	1867
CT_R	CACAGTTTGTGTGGAATCGG		
lci16_a_F	CAGGGGAGTAGCAAAACAG	*lci16-1*, *lci16-2*, *lci16-3*	1643
lci16_a_R	GCTGCTTCATGTGACCTTG		
pme1_a_F	CATAGAGCTTGCCATGTTATATCC	*pme1-1*, *pme1-3*	1025
pme1_a_R	AGAAGGAGACGAAAGCACAGG		
pme1_b_F	GGCTTGGATGACTGATGACCG	*pme1-2*	1014
pme1_b_R	ACCTCGCCAATGCACAGACG		

Supporting Information Table S1

Primers used for PCR verification of the *Chlamydomonas reinhardtii lci16* and *pme1* insertional mutant alleles.

References

Kafri M, Patena W, Martin L, Wang L, Gomer G, Ergun SL, Sirkejyan AK, Goh A, Wilson AT, Gavrilenko SE, *et al.* 2023. Systematic identification and characterization of genes in the regulation and biogenesis of photosynthetic machinery. *Cell* **186**: 5638-5655.e25.

Kyte J, Doolittle RF. **1982**. A simple method for displaying the hydropathic character of a protein. *Journal of Molecular Biology* **157**: 105–132.

Strenkert D, Schmollinger S, Gallaher SD, Salomé PA, Purvine SO, Nicora CD, Mettler-Altmann T, Soubeyrand E, Weber APM, Lipton MS, *et al.* 2019. Multiomics resolution of molecular events during a day in the life of Chlamydomonas. *Proceedings of the National Academy of Sciences* **116**: 2374–2383.
